# The optimal scaffold for silk sericin-based bone graft: collagen versus gelatin

**DOI:** 10.1186/s40902-022-00368-0

**Published:** 2023-01-09

**Authors:** Ji Hae Lee, HaeYong Kweon, Ji-Hyeon Oh, Seong-Gon Kim

**Affiliations:** 1 Sericultural and Apicultural Division, National Institute of Agricultural Science, RDA, Wanju, 55365 Republic of Korea; 2 Department of Oral and Maxillofacial Surgery, College of Dentistry, Gangneung-Wonju National University, Gangneung, 28644 Republic of Korea

**Keywords:** Silk sericin, Collagen, Gelatin, Scaffold, Bone graft

## Abstract

**Background:**

Silk sericin is an active ingredient in bone grafts. However, the optimal scaffold for silk sericin has yet to be identified.

**Method:**

A critical-sized bone defect model in rat calvaria was used to evaluate bone regeneration. Silk sericin from *Yeonnokjam*, *Bombyx mori*, was incorporated into gelatin (group G, *n* = 6) and collagen (group C, *n* = 6). Bone regeneration was evaluated using micro-computed tomography (mCT) and histology.

**Results:**

Group C showed a larger bone volume than group G in the mCT analysis (*P* = 0.001). Histological analysis showed a larger area of bony defects in group G than in group C. The bone regeneration area in group C was significantly larger than that in group G (*P* = 0.003).

**Conclusion:**

Compared with gelatin, collagen shows better bone regeneration in silk sericin-based bone grafts.

## Background

Silk sericin is an industrial waste produced by degumming. The silkworm cocoon is mainly composed of silk fibroin and silk sericin [1]. Silk sericin is bonding protein between silk fibers [2]. As silk sericin is mostly composed of hydrophilic amino acid such as serine, it is easily undergone hydrolysis as fragmented form [3]. Silk sericin has been used in cosmetics and burn dressings [1, 2] reducing unnecessary industrial waste. Silk mat-associated bone regeneration is mediated by released silk sericin from its surface [4]. Accordingly, silk sericin can be considered to have an osteo-induction property, and this is mediated by the activation of bone-resident macrophage [5].

Bone morphogenic protein-2 (BMP-2) is a potent bone inducer in the transforming growth factor-*β* superfamily [6]. BMP-2 can induce new bone formation both orthotopically and ectopically [7]. Recombinant human BMP-2 (rhBMP-2) is synthesized from microorganism or cell, and it has been widely used for bone tissue engineering. Because of the early loss of rhBMP-2 at the application site, a very high dose is required for bone regeneration [8]. In addition to the high cost of rhBMP-2, its direct application may result in postoperative complications such as severe inflammation [9], neoplasm [10], and ectopic bone formation [4]. Since silk sericin can induce BMP-2 expression in macrophages, applying it to bony defects can accelerate bone regeneration in a physiological manner, and it is much cheaper than rhBMP-2 [5]. Because silk sericin is primarily a hydrophilic protein, a scaffold may be required for its application to bony defects.

A collagen plug can be applied to the extraction socket. Collagen plugs are primarily used for ridge preservation after tooth extraction [11]. Because type 1 collagen is the primary component of the bone matrix, acellular type 1 collagen has been widely used as a scaffold for rhBMP-2, with generally acceptable results [12, 13]. Gelatin is also a component of the extracellular matrix. Gelatin sponges have been used to control bleeding. The application of gelatin sponges to tooth extraction sockets can prevent bleeding-associated complications [14]. Gelatin sponges have also been used as a scaffold [5]. However, direct comparisons of collagen and gelatin as scaffolds for bone tissue engineering are rare.

The objective of this study was to compare collagen and gelatin sponges as silk sericin scaffolds. The silk sericin was extracted from *Yeonnokjam*. They are a subspecies of *Bombyx mori* from Korea. *Yeonnokjam* has highest antioxidant activity among other Korea bred silkworms such as *Bakokjam*, *Golden silk*, and *Hanseongjam* [15]. Both collagen and gelatin were treated with silk sericin. These grafts were applied to calvarial defects in rats. Bone regeneration was evaluated using micro-computed tomography (mCT) and histology.

## Methods

### Sericin preparation

**Fig. 1:**
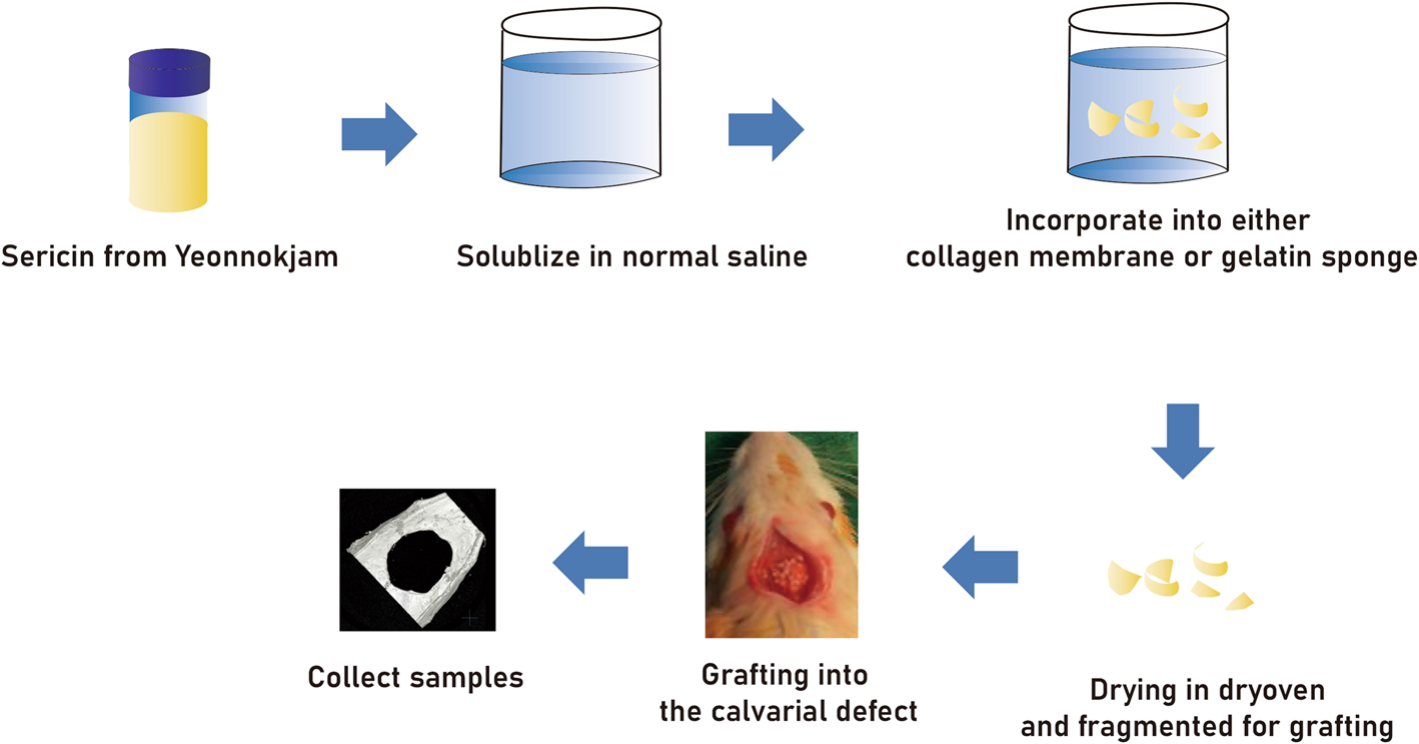
Schematic drawings of experimental procedure

*Yeonnokjam* was prepared by the Sericultural and Apicultural Division, National Institute of Agricultural Science, Rural Development Administration (RDA). The sericin from *Yeonnokjam* was treated by 4-hexylresorcinol (4HR) before degumming process to improve its protein conformation [16]. Gelatin sponge (Cutanplast Dental®, Uniplex, Sheffield, UK) with *Yeonnokjam* sericin (group G) and collagen membrane (Geistlich Bio-Gide®, Geistlich Pharma AG, Switzerland) with *Yeonnokjam* sericin (group C) were used as graft materials. All prepared grafts were fragmented for implanting into the bony defect. The detailed sample preparation procedure was shown in Fig. 1. Each graft contained approximately 50 μg of sericin.

### Animal experiment

We used Sprague-Dawley rats purchased from Samtako Inc. (Osan, Korea). The animal experiments were approved by the Gangneung-Wonju National University for Animal Research (GWNU-2022-7). Twelve rats that had been housed individually were divided into two groups: group G and group C. Group G (*n* = 6) was treated with a gelatin sponge containing sericin from *Yeonnokjam*, whereas group C (*n* = 6) was treated with a collagen membrane containing sericin from *Yeonnokjam*. All animals were given general anesthesia for the procedure using a combination of 0.5 mL Zoletil™ (125 mg/mL; Bayer Korea, Seoul, Korea) and 0.5 mL of Rompun^®^ (100 mg/mL; Bayer Korea, Seoul, Korea). Each animal received 0.3 mL of the anesthetic solution. The subsequent surgical procedures were carried out under aseptic conditions. Following the calvarial skin incision, a critical-sized defect was created in the calvaria with a trephine bur (diameter: 8.0 mm). The calvarial bone was removed, and its periosteum was covered. Grafts were randomly assigned to each animal. After grafting into the bony defect, the flap was closed in a single layer with 3-0 black silk. To prevent postoperative pain and infection, gentamicin (5 mg/day) and tolfenamic acid (4 mg/day) were injected intramuscularly for 2 days postoperatively. All animals were monitored for 8 weeks following surgery. After 8 weeks, all rats were sacrificed, and calvaria specimens were processed for further analysis.

### Micro-computed tomography (mCT) and histological analysis

Calvarial samples were analyzed using a μCT50 (Scanco Medical, Brüttisellen, Switzerland) at the Center for Research Facilities at Gangneung-Wonju National University. The size of the bony defect (8-mm-diameter circle) originally prepared determined the region of interest (ROI). The ROI bone volume (BV) was measured using mCT software (CT Analyzer V.1.17.7.2+, Skyscan), with the grayscale threshold set between 48 and 255.

The extracted bony specimens were fixed in 4% formaldehyde overnight at 4 °C. After washing the fixed samples with tap water, they were placed in a decalcification solution (Cat no.: MKCL9701, Sigma-Aldrich). The samples were then placed on a rocking plate for 3–5 days. Decalcified tissues were processed in a tissue processor before being embedded in paraffin. The thickness of the paraffin sections was 5 μm. Tissue sections in the midsagittal area were stained with hematoxylin and eosin. The histological images were captured using a light microscope (BX51, Olympus, Tokyo, Japan). The area of bone regeneration in the midsagittal area was analyzed at ×20 magnification. The ROI was determined based on the original defect size (length: 8 mm). Within the ROI, the area of bone regeneration was measured using image analysis software (Sigma Scan Pro 5.0, SPSS, Chicago, IL, USA). Additionally, immunostaining with BMP-2 antibody (CAT# sc-137087, SantaCruz Biotechnology, Santa Cruz, CA, USA). Subsequent procedure was in accord to our previous publication [16].

### Statistical analysis

The level of significance was set at *P* < 0.05. The independent sample *t*-test was used to compare the groups.

## Results

**Fig. 2:**
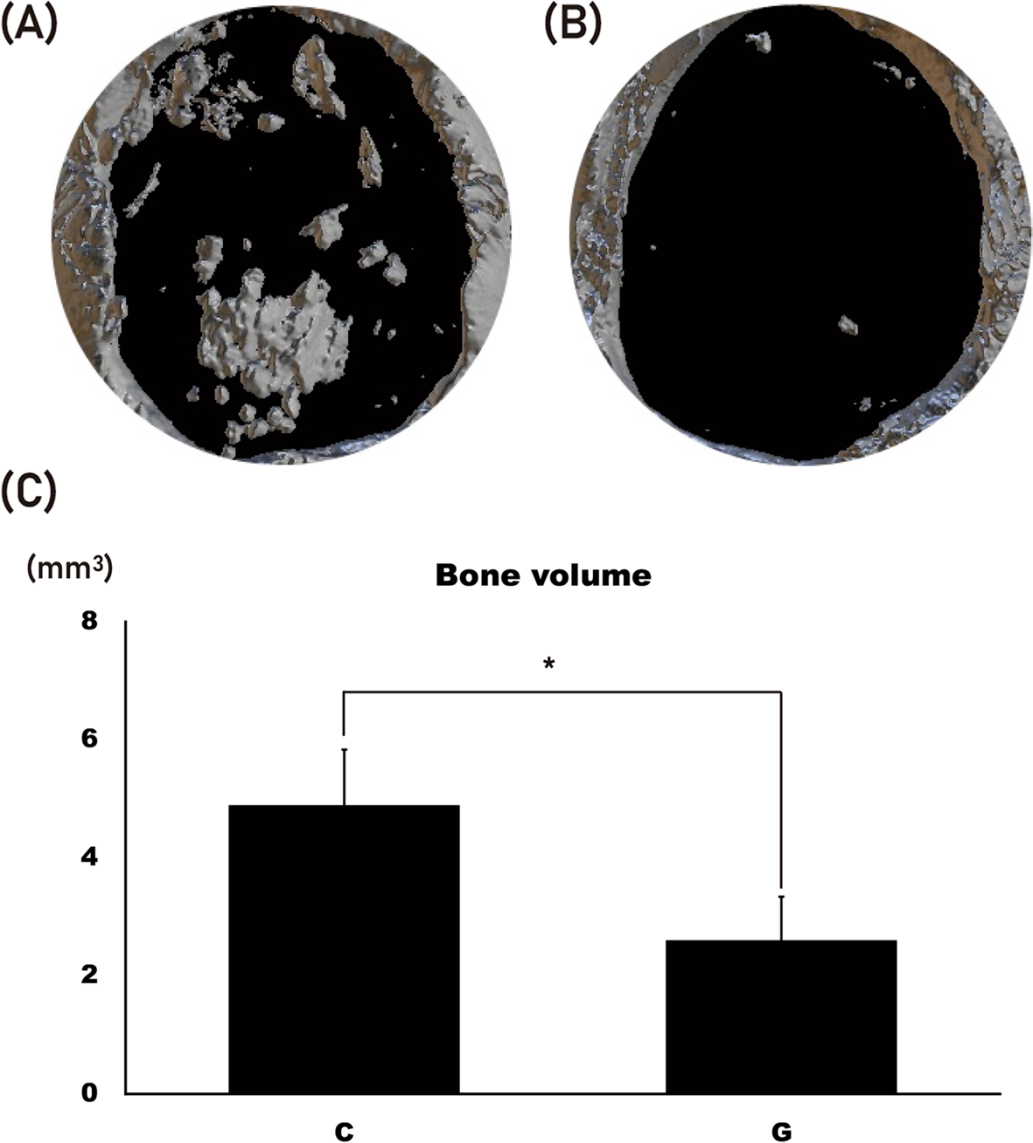
The results from micro-CT analysis. **A** The collagen group (group C) showed multiple calcified nodules in the bony defect. **B** The gelatin group (group G) also showed bone regeneration. However, it was less active compared with the collagen group. **C** Bone volume (BV) was measured in the region of interest. BV was significantly higher in group C than in group G (*P = 0.001)

**Fig. 3:**
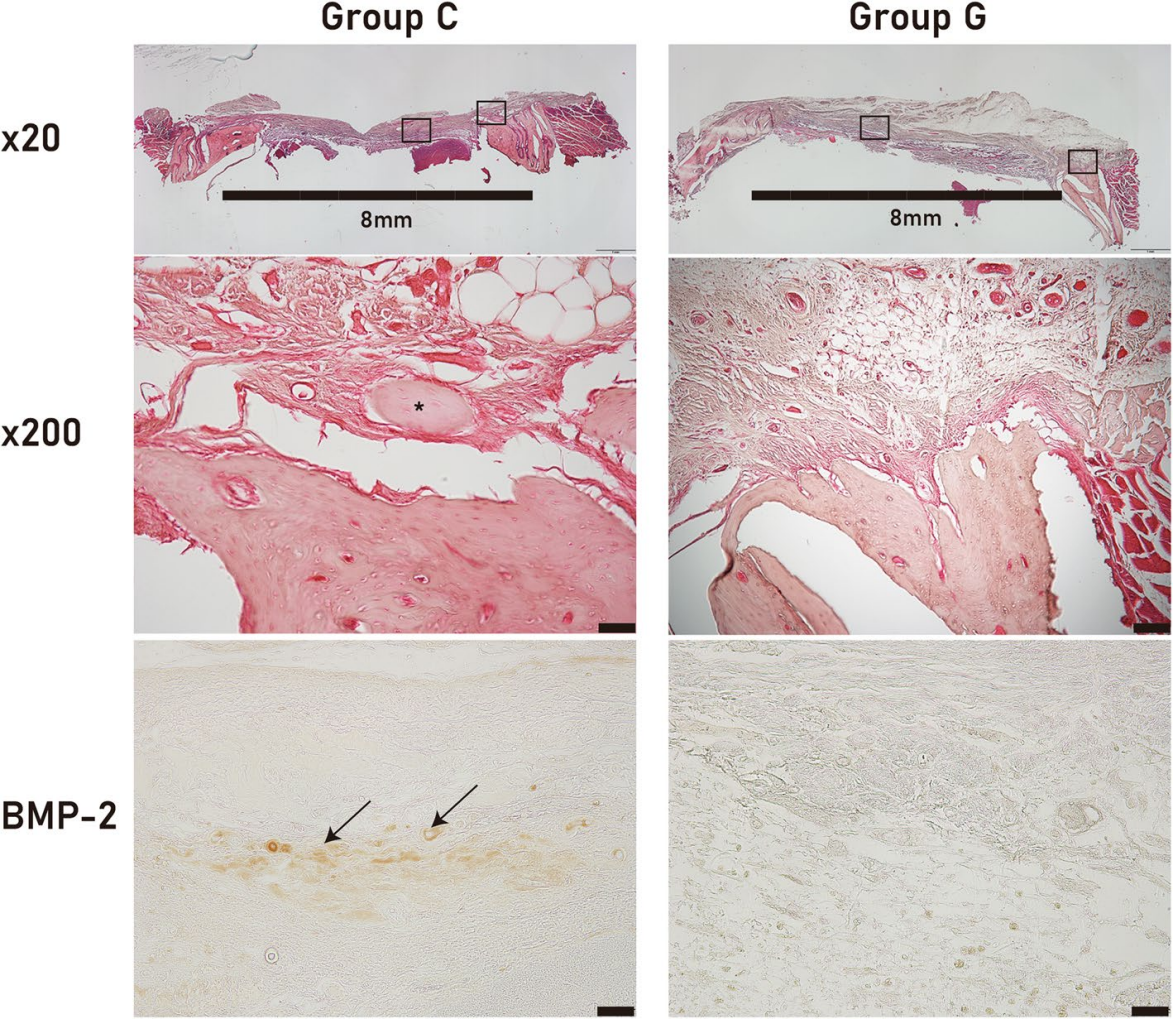
Histological analysis. The original bony defect had been prepared as 8 mm (bar in low magnification view). The residual bony defect area at 8 weeks postoperatively in the collagen group (group C) was smaller than in the gelatin group (group G). Bone regeneration from the margin was examined with high magnification view. The view of group C at high magnification showed a regenerated bony island (*). The view of group C under high magnification also showed bone ingrowth into the defect (hematoxylin and eosin stain, bar in high magnification = 50 μm). The expression level of BMP-2 was observed in the middle of bony defect. The expression level of BMP-2 was also higher in group C than in group G

**Fig. 4:**
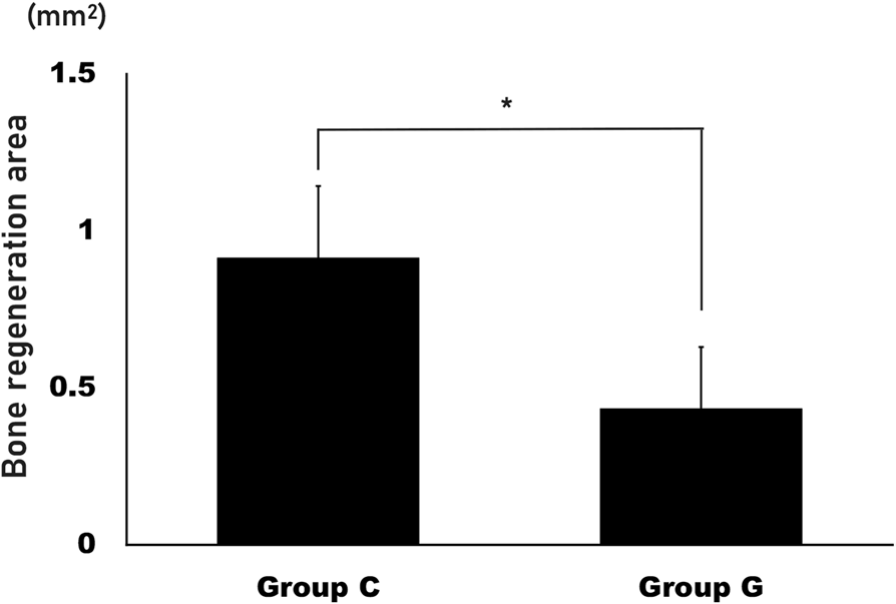
Histological analysis of bone regeneration area. Based on the defect size, the area of bone regeneration was measured and compared between the collagen group (group C) and the gelatin group (group G). The area of bone regeneration in group C was significantly larger than in group G (*P = 0.003)

The collagen sponge incorporated with the *Yeonnokjam* sericin (group C) (Fig. 2a) demonstrated greater bone regeneration than the gelatin sponge incorporated with the *Yeonnokjam* sericin (group G) (Fig. 2b). The difference in BV between the two groups was significant (*P* = 0.001), with group C having a BV of 4.89 ± 0.95 mm^3^ and group G having a BV of 2.60 ± 0.75 mm^3^ (Fig. 2c). Histological analysis revealed that group G had a larger area of bony defect than group C (Fig. 3). The expression level of BMP-2 was higher in group C than in group G (Fig. 3). The area of bone regeneration differed significantly (*P* = 0.003) between the groups, with group C having a bone regenerated area of 0.91 ± 0.23 mm^2^ (Figs. 4) and group G having a bone regenerated area of 0.43 ± 0.20 mm^2^.

## Discussion

Direct application of BMP-2 has been linked to a number of issues, such as swelling, pain, and ectopic bone formation [4, 8, 9]. By activating macrophages, silk sericin can accelerate bone healing [5]. An appropriate scaffold is required for the controlled release of silk sericin during application. Collagen and gelatin have both been used as scaffolds for bone tissue engineering. In this study, both scaffolds were compared for use in silk sericin-based bone grafts. In the mCT analysis, BV was significantly larger in group C than in group G (Fig. 2; *P* = 0.001). Histological analysis also confirmed that group C had better bone regeneration (Figs. 3 and 4). This is to the best of our knowledge, the first comparative study of different silk sericin scaffolds.

Silk sericin is the main component of silkworm cocoons which shows osteogenic properties by activating macrophages [5]. When silk mat is grafted into a bone defect, macrophages migrate into the graft as a result of the foreign body response [17]. The silk sericin released from the surface of the silk mat is recognized by bone resident macrophages via toll-like receptors [5]. Subsequently, these macrophages release BMP-2 to recruit bone forming cells such as osteoblasts. When sericin is isolated via the degumming process, the sericin + scaffold combination graft can accelerate bone healing [16]. The advantages of silk sericin usage for bone tissue engineering is (1) reducing industrial waste, (2) cheaper price compared to rhBMP-2, and (3) less complications expected compared to direct application of rhBMP-2 [5]. The disadvantages of silk sericin is (1) different level of bone regeneration performance to degumming techniques [5], (2) different level of biological performance to its species [15], and (3) difficulty in quality control during mass production compared to synthetic one. When silkworm cocoon is treated by 4HR, *β*-sheet structure of degumming product is increased, and it is helpful for bone regeneration [16].

Scaffolds are an important component of tissue engineering. For the rhBMP-2 scaffold, collagen, chitosan, gelatin, silk fibroin, dextran, and hyaluronic acid have been investigated [18]. Collagen has been frequently used as the scaffold for rhBMP-2 owing to its biological inertness, low foreign body reaction, and controlled degradation via cross-linking [19, 20]. The pitfalls of collagen as a scaffold are its rapid degradation, low mechanical strength, and rapid release of active ingredients [21, 22]. However, manufacturing techniques such as cross-linking can be used to mitigate these flaws [23]. In this study, type 1 collagen membrane for the guided bone regeneration was used as a scaffold for sericin because this collagen is cross-linked. A cross-linked collagen matrix is excellent for the growth of the osteoprogenitor cells [12]. As only 2 types of scaffolds had been tested in this study, other scaffold might show better performance for sericin. Considering the importance of a suitable scaffold, different scaffold types for silk sericin should be investigated.

In this study, collagen and gelatin scaffolds for silk sericin were compared. The sericin incorporated into collagen demonstrated better bone regeneration than gelatin (Figs. 2 and 3). Gelatin can immobilize rhBMP-2 and release it slowly in a controlled manner [24]. In some cases, rhBMP-2 is first incorporated into gelatin, which is then mixed with chitosan/collagen scaffold [25]. Gelatin has an advantage as a protein carrier owing to its protein stabilizing ability and sustained release [26]. The high porosity of gelatin facilitates protein absorption [27]. However, due to its rapid degeneration, gelatin sponge alone may not be an ideal scaffold for bone graft [28]. BMP-2 has a positive surface charge and has a high binding affinity for negatively charged heparin [29]. Using this characteristic of BMP-2, poly-glutamic acid was utilized to enhance rhBMP-2-associated osteogenesis [30]. The surface charge of silk sericin has not yet been investigated, but there could be a difference between how it interacts with collagen and gelatin. This difference may affect how they bind to silk sericin when used as scaffolds.

This study has a number of limitations. First, a number of characteristics could impact the performance of scaffolding. Even for collagen, pore size, cross-linking method, and source can affect a tissue’s regenerative ability [22, 23]. Second, there are multiple subspecies of *Bombyx mori* [31]. In our preliminary study, the sericin from different subspecies induced BMP-2 in macrophages to varying degrees (data not shown). As the amino acid sequence of the same type of protein can vary between subspecies, its binding affinity to the target protein will also vary. Third, as this was a preliminary study, the number of animals in this study was relatively small. There may be differences in the capacity for healing among experimental animals. Although there was a significant difference between groups in this study, considering the aforementioned points, further confirmative studies should be conducted.

## Conclusion

In this study, two different scaffold types for silk sericin-based bone graft were evaluated. Collagen demonstrated superior bone regeneration for silk sericin-based bone graft when compared with gelatin.

## Data Availability

Data sharing is not applicable to this article as no datasets were generated or analyzed during the current study.

## Abbreviations

4HR: 4-Hexylresorcinol
BMP-2: Bone morphogenic protein-2
mCT: Micro-computed tomography
ROI: Region of interest
